# Publisher Correction: High mitogenic stimulation arrests angiogenesis

**DOI:** 10.1038/s41467-019-10474-9

**Published:** 2019-05-30

**Authors:** Samuel Pontes-Quero, Macarena Fernández-Chacón, Wen Luo, Federica Francesca Lunella, Verónica Casquero-Garcia, Irene Garcia-Gonzalez, Ana Hermoso, Susana F. Rocha, Mayank Bansal, Rui Benedito

**Affiliations:** Molecular Genetics of Angiogenesis Group, Centro Nacional de Investigaciones Cardiovasculares (CNIC), Madrid, 28029 Spain

**Keywords:** Cell signalling, Angiogenesis, Cell proliferation

Correction to: *Nature Communications* 10.1038/s41467-019-09875-7, published online 01 May 2019.

The original version of this Article contained errors in Fig. 8. In panel a, the labels ‘VEGF’, ‘Notch’, ‘p21’, and ‘P-ERK’ were inadvertently omitted. This has been corrected in the PDF and HTML versions of the Article.

