# Supplementary material for: High mitogenic stimulation arrests angiogenesis
Source: Nat Commun. 2019 May 1;10:2016. doi: 10.1038/s41467-019-09875-7 (PMC6494832; doi:10.1038/s41467-019-09875-7)
Supplement: Supplementary file 1 — Supplementary Information [file 41467_2019_9875_MOESM1_ESM.pdf]

Supplementary Information

High mitogenic stimulation arrests angiogenesis

Samuel Pontes-Quero, Macarena Fernández-Chacón, Wen Luo, Federica Lunella, Verónica Casquero-Garcia, Irene Garcia-Gonzalez, Ana Hermoso, Susana F. Rocha, Mayank Bansal, Rui Benedito.

It includes;
Supplementary Figures 1-8 and Supplementary Table 1.

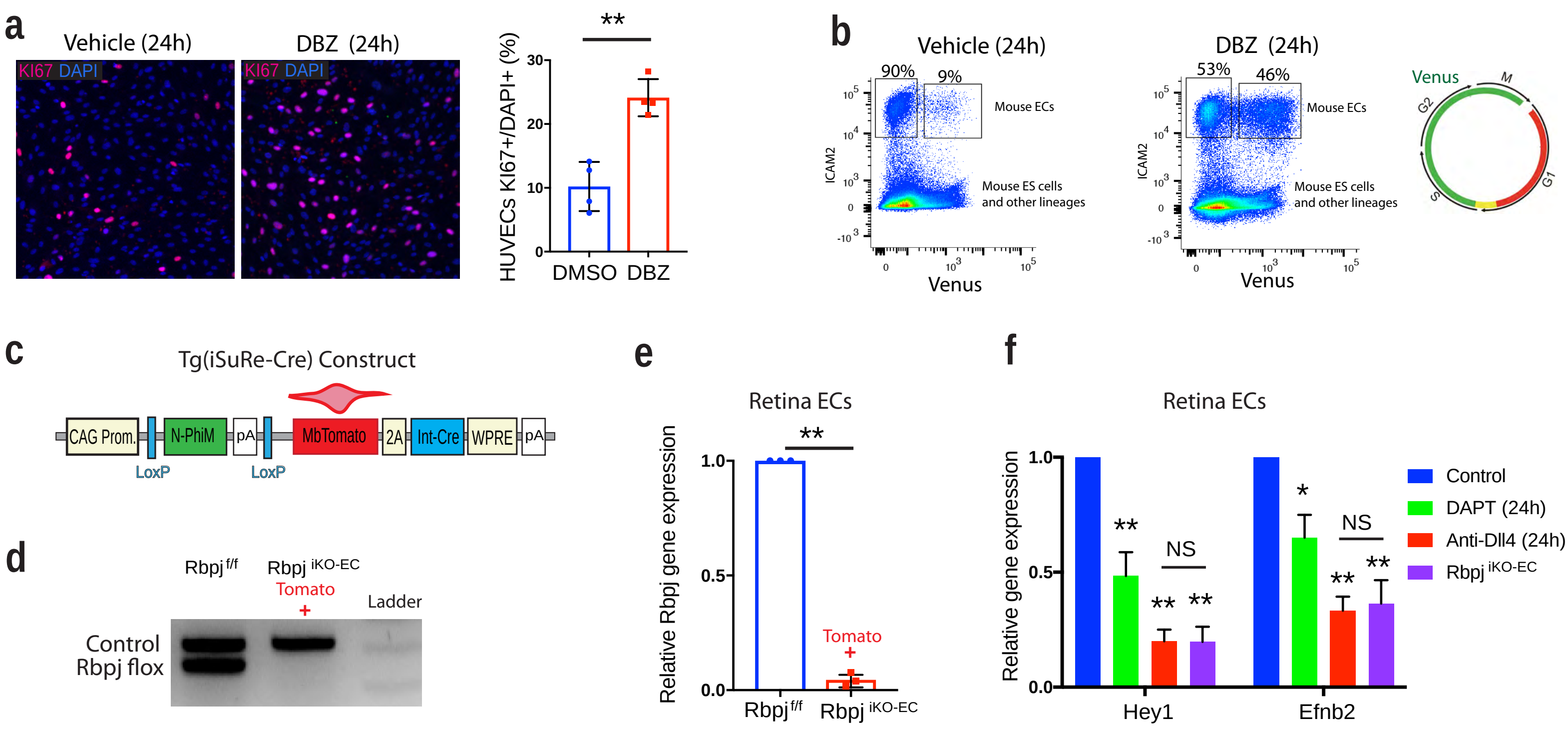

Supplementary Fig. 1 Proliferative effect of Notch inhibition *in vitro* and validation of different pharmacological and genetic approaches used to interfere with Notch signalling.
a) Inhibition of Notch signalling with DBZ for 24h increases the frequency of KI67+ HUVECs. n=4 independent experiments.
b) Inhibition of Notch signalling with DBZ for 24h in ECs derived from embryonic stem cells carrying a Rosa26knock-in FUCCI allele, increases the frequency of ECs (ICAM2+) in S/G2/M (Venus+).
c) Diagram representing the iSuRe-Cre allele. After inducible CreERT2 recombination, cells express MbTomato and a intron containing Cre.
d) Detection of the Rbpj floxed allele by PCR shows that deletion of the allele is complete in MbTomato+ cells collected by FACS from Rbpj^{iKO-EC} mutants.
e) qRT-PCR of RNA collected from CD31-APC+ Retina ECs of Rbpj$^{f/f}$ (n=3) or Rbpj^{iKO-EC} mutants (n=3), shows the pronounced reduction in Rbpj mRNA levels in MbTomato+ cells.
f) Comparison by qRT-PCR of canonical endothelial Notch targets (Hey1 and Efnb2) expression in retina ECs collected by FACS from animals receiving DAPT (for 24h, n=3) or Anti-Dll4 (for 24h, n=3) or with induced *Rbpj* deletion for 5 days (n=3). Error bars indicate StDev; NS, non-significant; * $p < 0.05$; ** $p < 0.005$ relative to control. Two-tailed unpaired T-test (a, e). One-way ANOVA with Tukey's post hoc test (f). Source data are provided as a Source Data file.

Supplementary Fig. 2 *Pontes-Quero et al.*

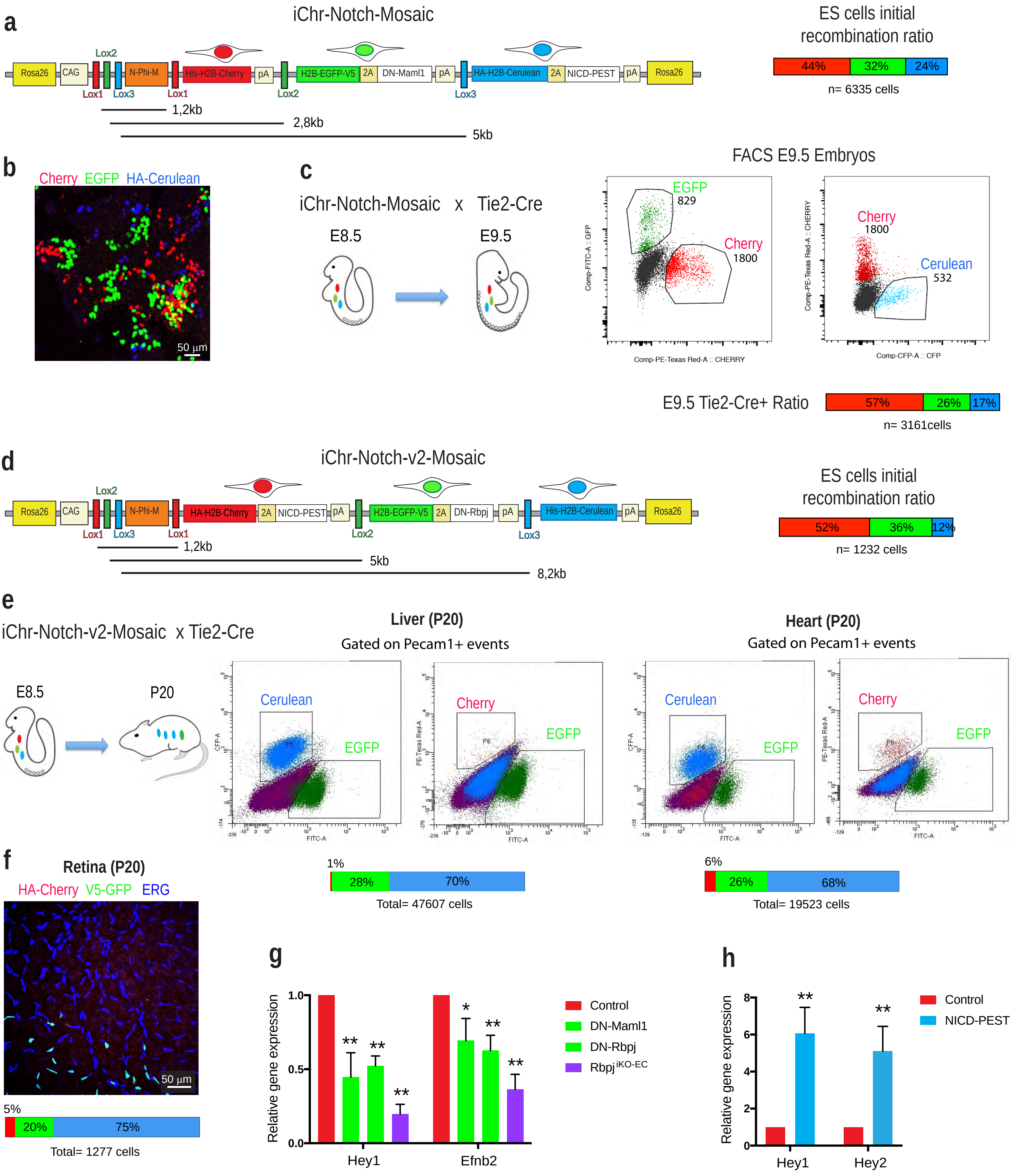

Supplementary Fig. 2 iChr-Notch-Mosaic constructs, mosaic ratios and canonical Notch target genes profiling.
a, b) Diagram of *iChr-Notch-Mosaic* allele, with the distances between loxP sites and obtained frequencies in mouse ES cells after Cre plasmid transfection.
c) Mice carrying the *iChr-Notch-Mosaic* and *Tie2-Cre* alleles have recombination of the construct between E8.0 and E8.5. FACS analysis of embryos at E9.5 (n=4) reveals the proportion of ECs expressing the different markers at that timepoint. Note the difference between ES cells (a) and E9.5 embryos (c).
d) The *iChr-Notch-V2-Mosaic* allele contains *DN-Rbpj* instead of *DN-Maml1*, and a change in the position of the control cassette (Cerulean+), resulting in the lower occurrence of these cells.
e, f) Mice carrying the *iChr-Notch-V2-Mosaic* and *Tie2-Cre* alleles will have recombination of the construct between E8.0 and E8.5. FACS analysis of animals at P20 reveals the proportion of ECs expressing the different markers at that timepoint. Note the difference between the initial recombination ratio in ES cells (d) and in the vasculature of the different organs (e, f).
g, h) Comparison by qRT-PCR of canonical Notch targets expression in ECs collected by FACS from *iChr-Notch-Mosaic* (n=3) and $Rbpj^{iKO\text{-}EC}$ animals (n=3).
Error bars indicate StDev; NS, non-significant; * p< 0.05; ** p<0.005. One-way ANOVA with Tukey's post hoc test (g) and Two-tailed unpaired T-test (h). Source data are provided as a Source Data file.

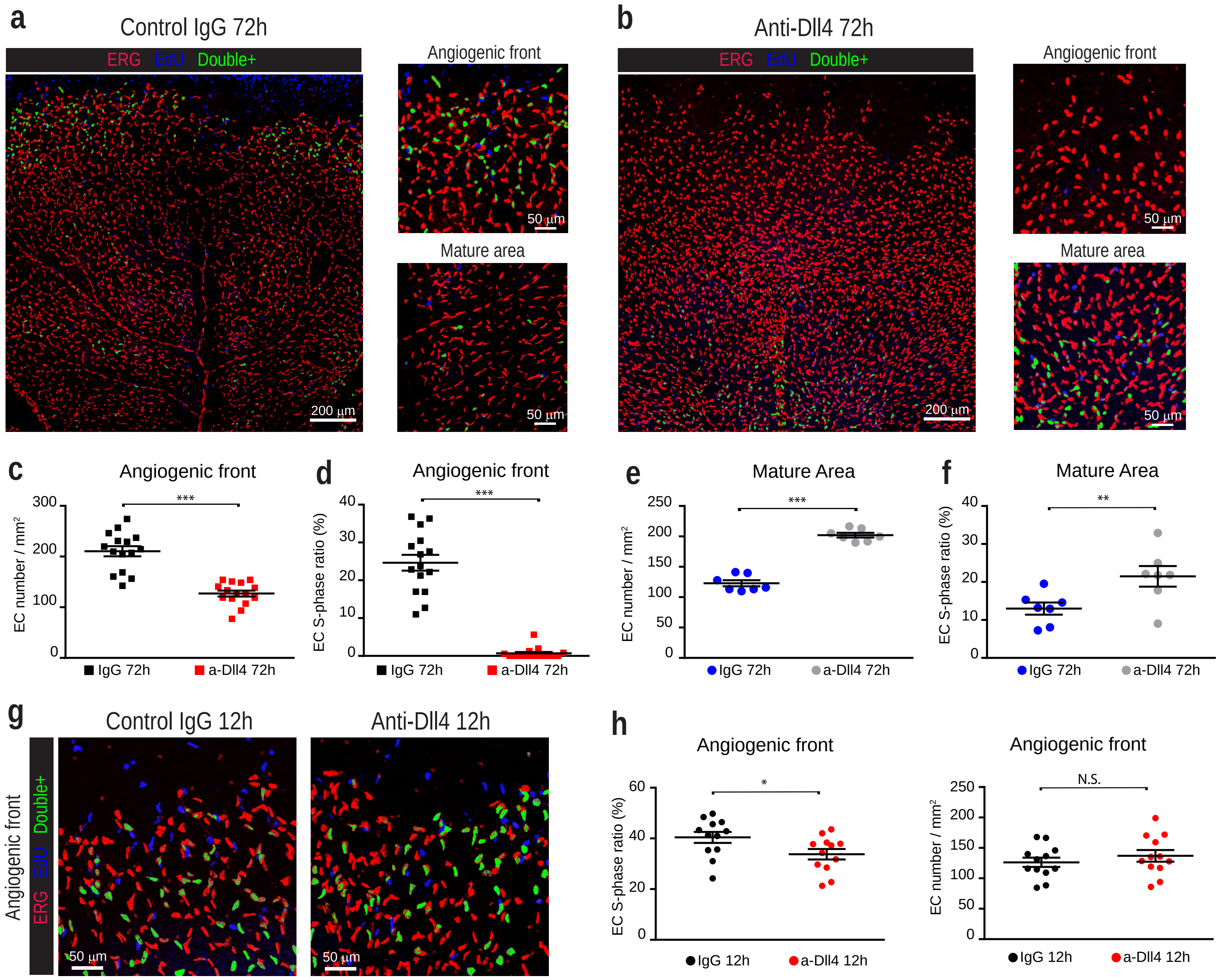

Supplementary Figure 3. Temporal analysis of the proliferative effect of Notch inhibition in angiogenic and mature vessels ***in vivo*****.**

a, b) Confocal micrographs of retinal vasculature from animals treated with IgG (control) and anti-Dll4 for 72h and immunostained with anti-Erg (red signal, labels EC nuclei) and with EdU labelling in the nuclei of all cells in S-phase in the 4 h before dissection. Blue signal is EdU in the nuclei of non-endothelial (ERG-) cells, and green signal indicates ERG+/EdU+ endothelial nuclei (ERG+ Red and EdU+ Blue signals when colocalize result in pink color, that was pseudocolored to green to better highlight ECs in S-phase. Higher magnification pictures are provided to show the distinct effects of Dll4/Notch inhibition in the proliferative angiogenic front and in the mature/quiescent area.

c-f) Charts showing that Dll4/Notch signaling inhibition with anti-Dll4 for 72h leads to premature cell cycle exit at the angiogenic front and cell cycle entry of quiescent/mature ECs, indicated by the changes in the frequency of Erg+ cells in S-phase (EdU+, green) observed in these two distinct areas. This results in an increased EC density in the mature vascular area.

g, h) Similar analysis in animals treated with anti-Dll4 for only 12h indicates that at this stage there is not a significant difference in EC density or the frequency of ECs in S-phase.

Charts show comparative analysis of large microscopy fields taken from 4 retinas per group. Error bars indicate SEM; NS, non-significant; * $p< 0.05$; ** $p<0.005$; *** $p<0.0005$. Two-tailed unpaired T-test. Source data are provided as a Source Data file.

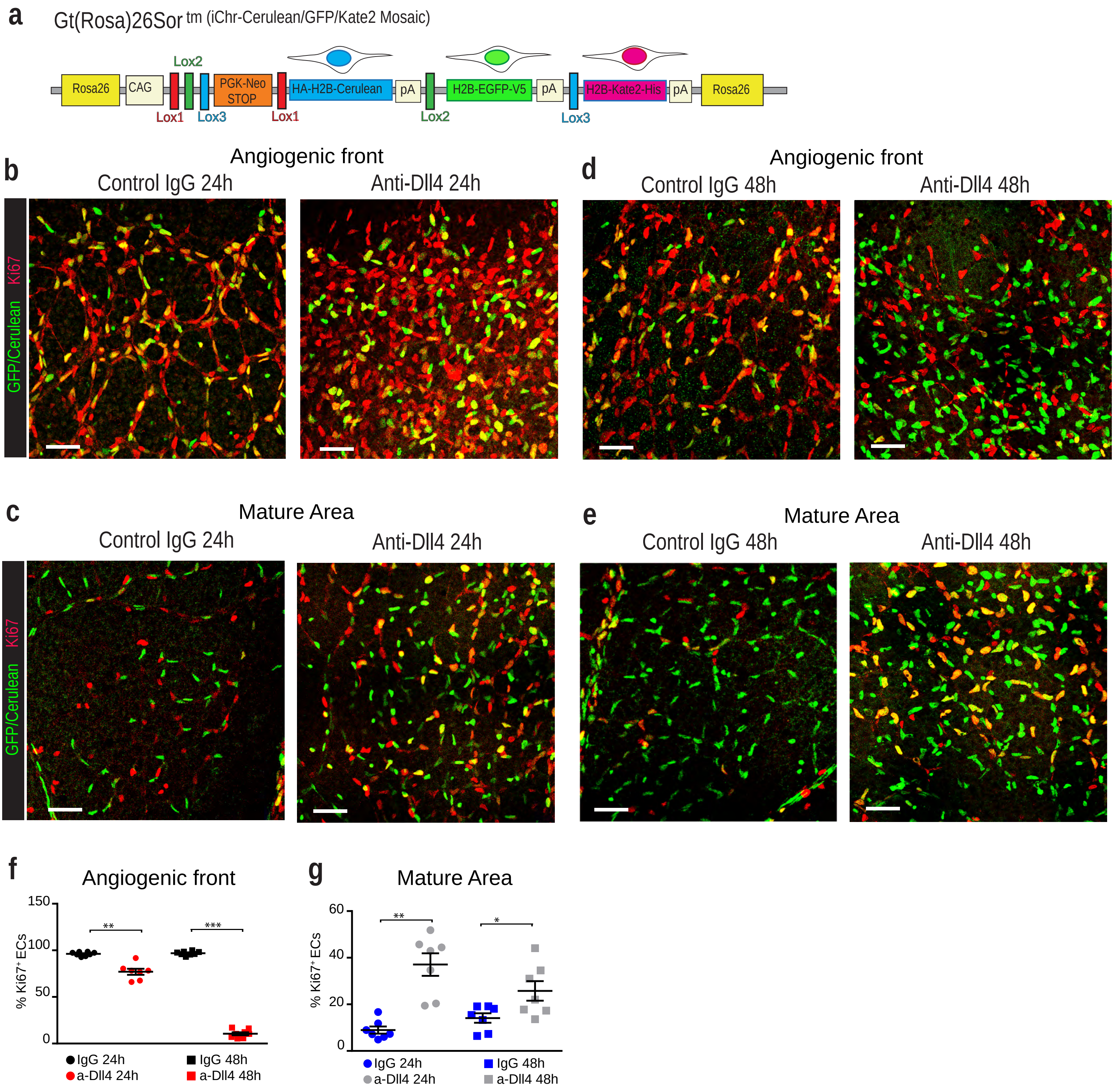

Supplementary Figure 4. Effect of Notch inhibition on the frequency of endothelial cells in cycle (Ki67+) in angiogenic and mature vessels.

a) Diagram illustrating the mouse *iChr-Cerulean/GFP* allele used to specifically label the nuclei of the endothelium with GFP and Cerulean proteins, in order to be possible to perform the co-immunostaining with rabbit anti-Ki67 and goat anti-GFP to detect the nuclei of ECs (we could not use the incompatible rabbit anti-ERG, that was combined with EdU in Sup. Fig. 3).

b-e) Confocal micrographs of retina vessels from animals carrying the *Tie2-Cre* and *iChr-Cerulean/GFP* reporter allele. In these mice, most ECs will express Cerulean or GFP in the nucleus, both recognized by the anti-GFP antibody. This allows detection in the same immunostaining of Ki67, present in all cycling cells. Yellow nuclei correspond to GFP+/Ki67+ cycling ECs and green nuclei to non-cycling ECs (GFP+/KI67-). Scale bars, 50µm.

f, g) Quantification of several microscopic fields as represented in b-e. Error bars indicate SEM; NS, non-significant; * $p < 0.05$; ** $p<0.005$; *** $p<0.0005$. One-way ANOVA with Tukey's post hoc test. Source data are provided as a Source Data file.

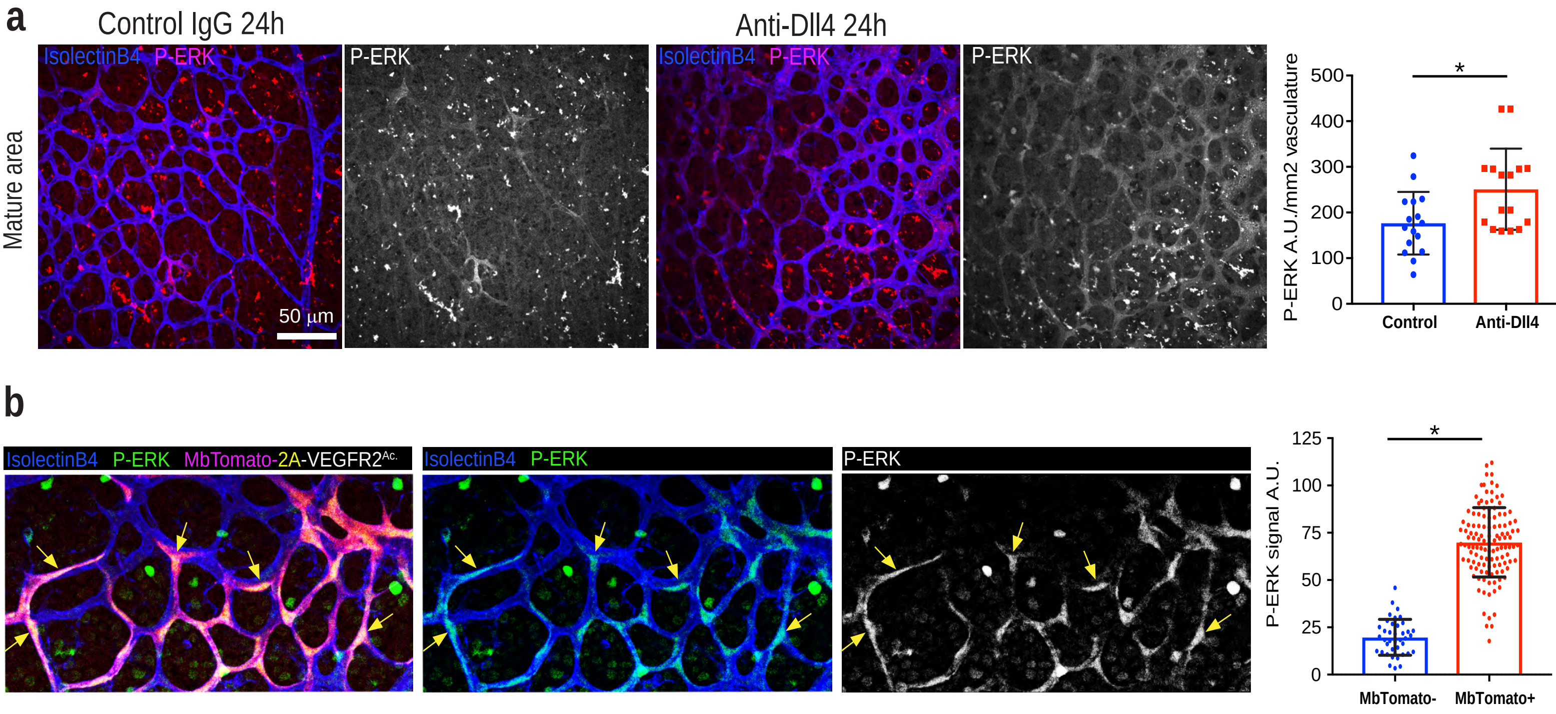

Supplementary Figure 5. ERK activity in mature ECs after Dll4/Notch inhibition or VEGFR2 activation.

a) Confocal micrographs of the mature retinal vasculature of control (n=3) and anti-Dll4 (n=4) treated animals, showing very low basal P-ERK levels in mature vessels (compare with Fig. 3a), that increase after Dll4/Notch signaling blockade for only 24h. However, P-ERK levels do not reach the very high level detected at the angiogenic front (compare with Fig. 3a right).

b) Confocal micrographs of P6 retina vessels from animals (n=3) carrying the *iMb-Vegfr2-Mosaic* and *Cdh5-CreERT2* alleles, three days after tamoxifen injection. Expression of *MbTomato-2A-Vegfr2Ac* (yellow arrows) induces a marked upregulation of P-ERK levels in endothelial cells. Each dot in the chart represents the average relative P-ERK signal in a single cell segmented area, in relation to the background signal in the IsolectinB4 negative area.

Error bars indicate StDev; * $p < 0.05$. Two-tailed unpaired T-test. Source data are provided as a Source Data file.

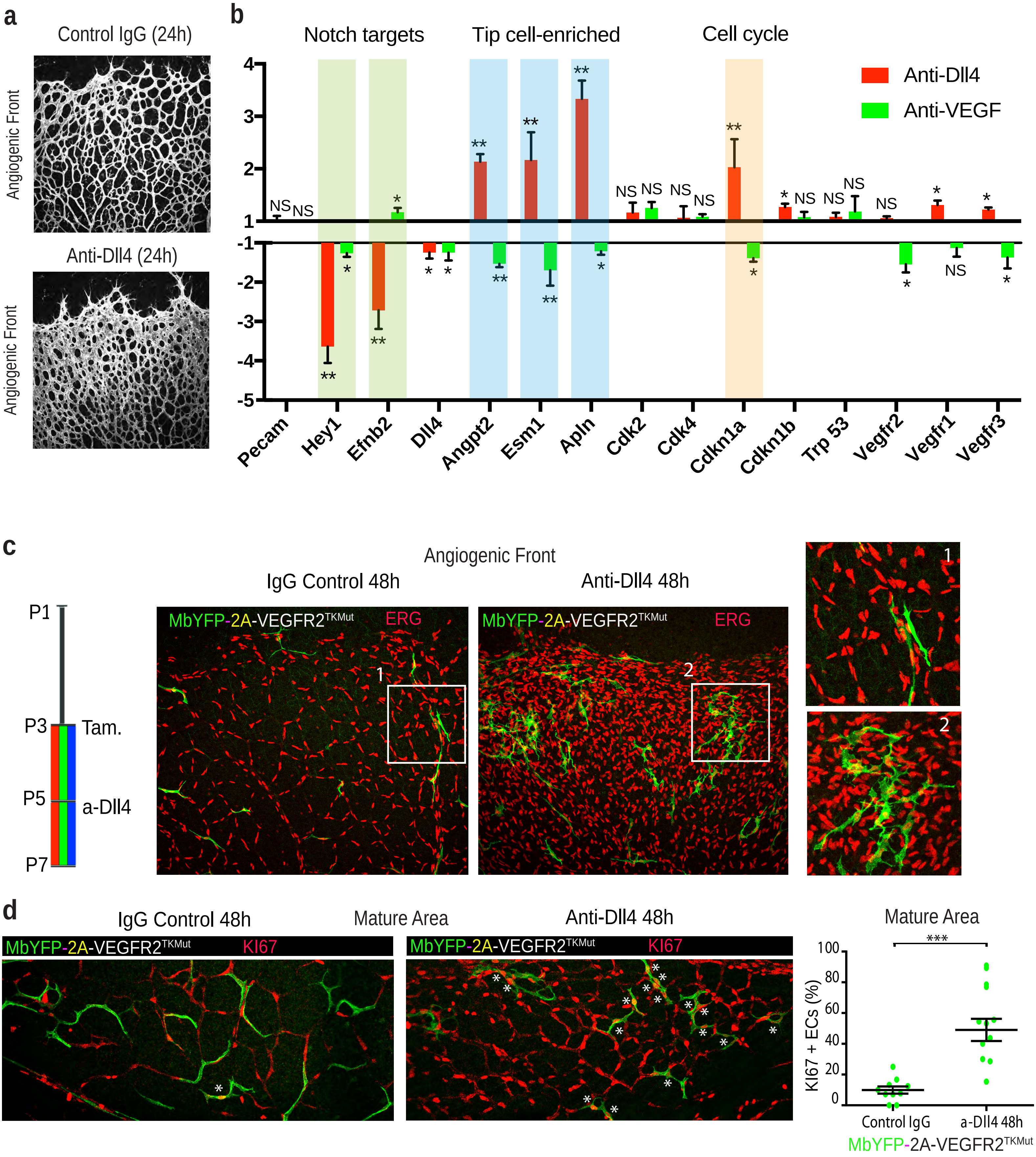
a
Control IgG (24h)
Angiogenic Front
Anti-Dll4 (24h)
Angiogenic Front
b
Notch targets
Tip cell-enriched
Cell cycle
Anti-Dll4
Anti-VEGF
Pecam
Hey1
Efnb2
Dll4
Angpt2
Esm1
Apln
Cdk2
Cdk4
Cdkn1a
Cdkn1b
Trp 53
Vegfr2
Vegfr1
Vegfr3
c
Angiogenic Front
IgG Control 48h
Anti-Dll4 48h
P1
P3
P5
P7
Tam.
a-Dll4
MbYFP-2A-VEGFR2TKMut ERG
d
IgG Control 48h
Mature Area
Anti-Dll4 48h
MbYFP-2A-VEGFR2TKMut KI67
Mature Area
KI67 + ECs (%)
Control IgG
a-Dll4 48h
MbYFP-2A-VEGFR2TKMut

Supplementary Fig. 6. Notch strongly represses the expression of tip cell enriched genes and Cdkn1a (p21) but not Vegfrs.

a) Confocal micrographs of the control and anti-Dll4 treated retinal vasculature stained with IsolectinB4.

b) Evaluation by qRT-PCR (n=3 independent experiments) of genes differentially expressed by angiogenic retina vessels after anti-Dll4 (n=6 animals) or anti-VEGF (n=6 animals) treatment for 24h. Tip cells enriched genes and Cdkn1a (p21) are significantly regulated by Notch and VEGF in a opposite manner.

c, d) Confocal micrographs of P7 retinas from *iMb-Vegfr2-Mosaic Cdh5-CreERT2* mice, 4 days after inducing with tamoxifen the expression of MbYFP-2A-VEGFR2TkMut in some ECs, and 2 days after receiving control IgG or Anti-Dll4 for 48h. When Dll4/Notch signaling is inhibited, VEGFR2TKMut expressing mature ECs enter the cell cycle (KI67+, asterisk) and are able to proliferate. Note that there is some unspecific KI67 signals in the membrane of some cells. In higher magnification panels shown in 1 and 2 is possible to see a difference in the morphology of MbYFP-2A-VEGFR2TkMut expressing cells induced by anti-Dll4 treatment.

Error bars indicate StDev in b and SEM in d; NS, non-significant; * $p< 0.05$; ** $p<0.005$; *** $p<0.0005$. Two-tailed unpaired T-test. Source data are provided as a Source Data file.

Supplementary Fig. 7

Pontes-Quero et al.

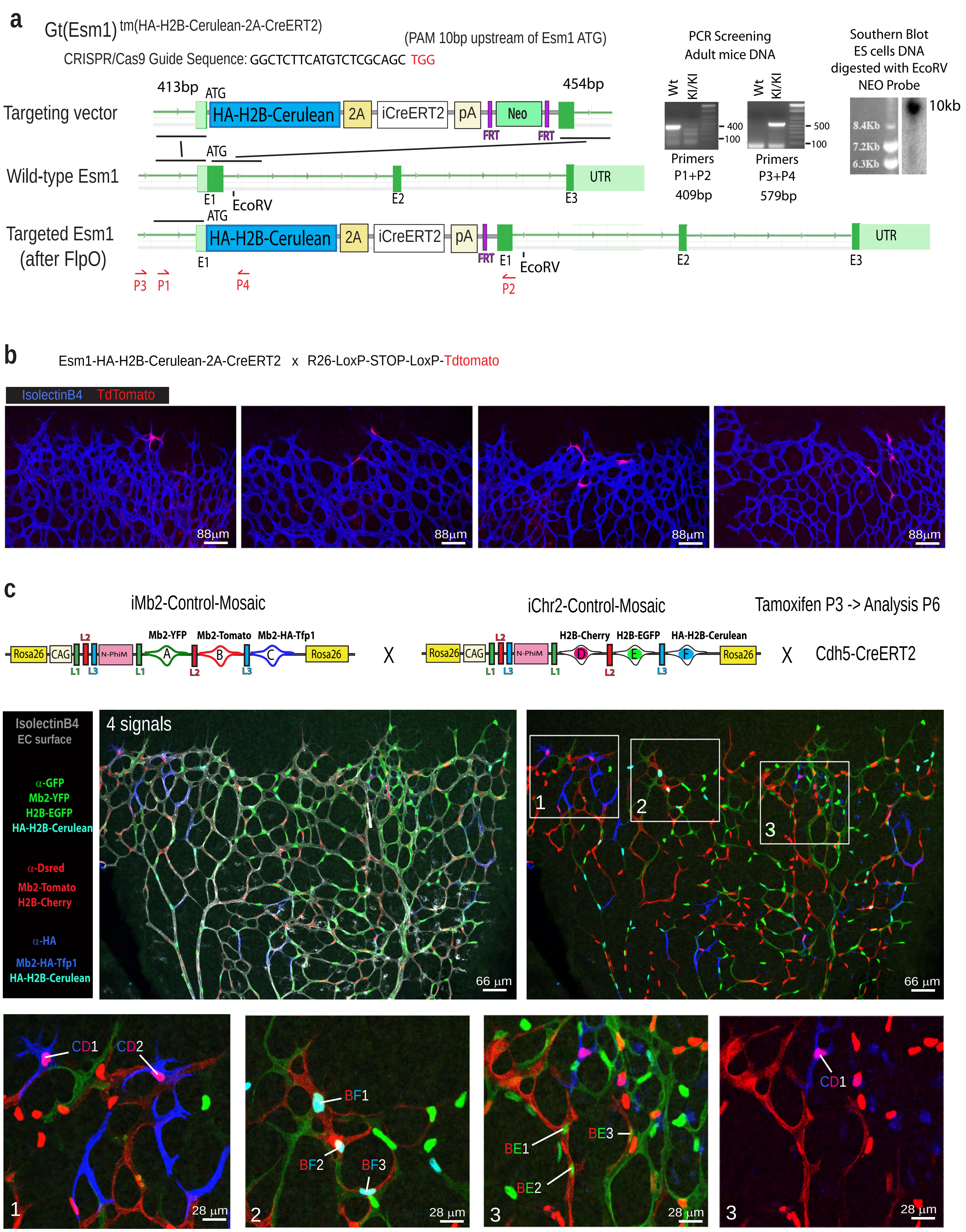

Supplementary Figure 7. A mouse line to label and genetically pulse ECs with high Esm1 expression.
a) Targeting vector and *Esm1* allele genetic maps. To label the nuclei and induce genetic recombination in individual endothelial tip cells, we introduced the *HA-H2B-Cerulean-2A-CreERT2-Sv40pA-FRT-Neo-FRT* cassette in the *Esm1* starting codon (ATG) by CRISPR/Cas9-induced double-strand break and homologous recombination-dependent repair. The donor vector contained two homology arms with 413 and 454bp, respectively, and was electroporated into ES cells. After selection in G418 (neomycin), the resistant clones were screened for correct vector integration, first by PCR with the indicated primers and after by Southern blot with a labeled probe against the Neo gene. The *FRT-Neo-FRT* cassette was later flipped out by transient transfection of FlpO, and clones without Neo resistant cells were used to generate mice.
b) Confocal micrographs from P7 retinas of mice with the above mentioned alleles and pulsed with tamoxifen at P3. In most retinas, there were either no labelled cells, or only 1 clone with labelled cells per flank (each dissected and flat-mounted retina has 4 flanks). Clones were of different sizes (see also Fig. 4c chart). Given the very low frequency of Tomato+ cells and their clonal and nearby distribution in the tissue, it is statistically highly improbable that clones with more than 2 cells arose from more than one independent stochastic tip-cell recombination event.
c) Representative confocal micrographs showing the 4 signals detected after immunostaining with the indicated antibodies, of the postnatal day 6 mouse retina of *Dual ifgMosaic* mice, carrying the *Cdh5-CreERT2* allele, and pulsed once with tamoxifen at P3. Magnified boxed areas (1 to 3) and two-letter codes show selected double-recombined cell clones and the observed combination of FPs A to F, at higher magnification. The chance of a given dual recombination event is lower than single recombination event, enabling the identification and quantification of single-cell derived clones (see also Pontes-Quero et al., 2017). This method was used for the quantifications shown in Fig. 4d chart.

Supplementary Figure 8

Pontes-Quero et al.

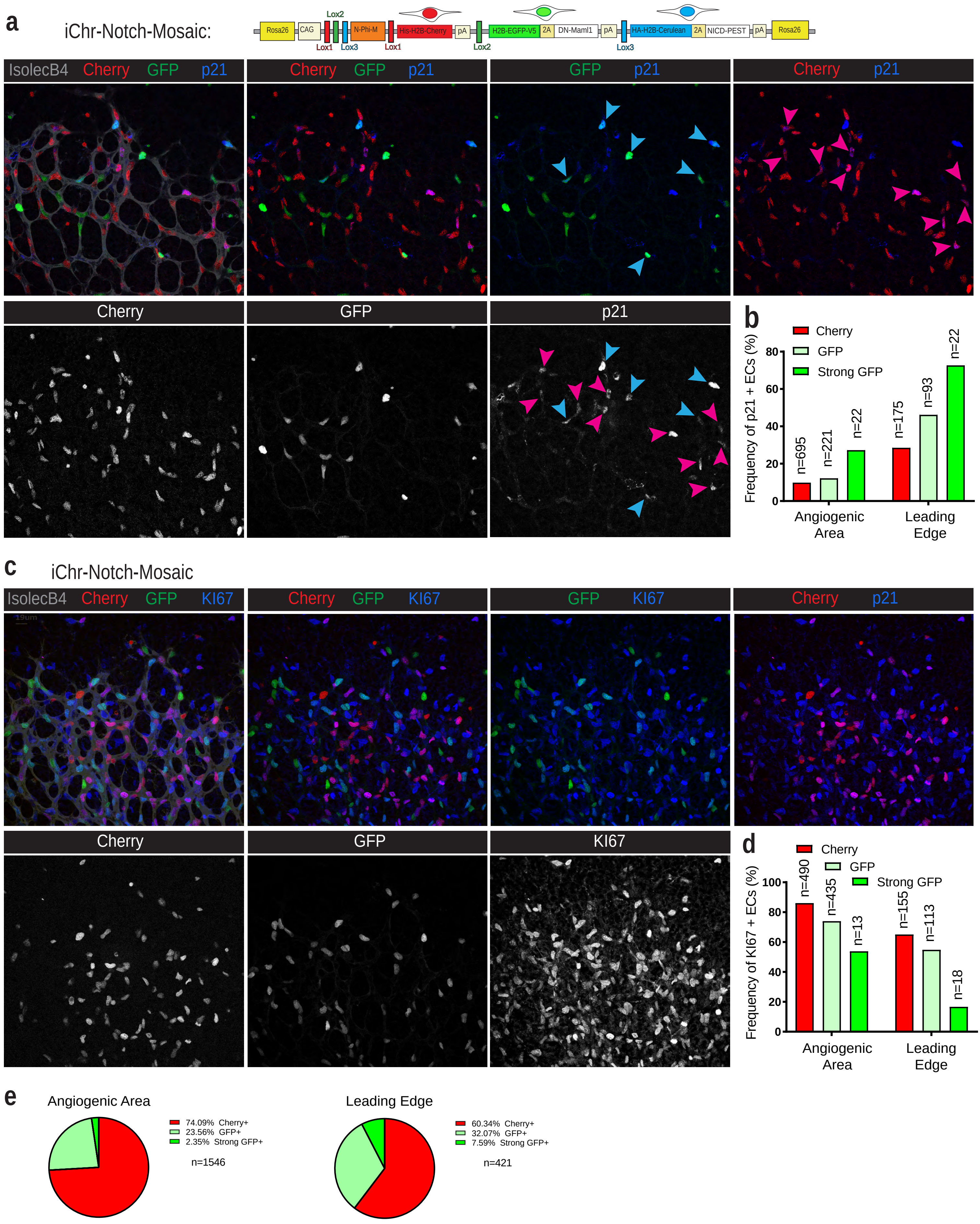

Supplementary Figure 8. Single cells with a decrease in Notch signalling exit cell cycle and sprout more frequently.
a, b) Confocal micrographs of the angiogenic front of P6 retinas from animals expressing the iChr-Notch-Mosaic allele in endothelial cells (IsolectinB4+). Individual cells with lower Notch signalling (GFP+) are more frequently p21+, especially if they express GFP strongly and are at the leading edge of the vessels. Blue arrowheads indicate GFP+/p21+ cells. Pink arrowheads indicate Cherry+/p21+ cells.
c, d) Confocal micrographs of the angiogenic front of P6 retinas from animals expressing the iChr-Notch-Mosaic allele in endothelial cells (IsolectinB4+). Individual cells with lower Notch signalling (GFP+) are less frequently KI67+, especially if they express GFP strongly and are at the leading edge of the vessels.
e) Cells with a decrease in Notch signalling (GFP+), are more frequently found at the leading edge of the vessels.

Supplementary Table 1

Pontes-Quero et al.

Primers used to genotype mice

Primer Name	Primer Sequence	PCR Ta	Band Size	Purpose
hVEGFR2 seq F	GCGGCACGAAATATCCTCT	60C	206bp	To detect the iMb-VEGFR2-Mosaic allele
hVEGFR2 seq R	ATTTCCCACAGCAAAACACC			
CTP PHI F	ACGTGAAGCTGAGCAAGGAT	60C	550bp	To detect the iChr-Mosaic allele
CTP H2B R	CTTAGTCACCGCCTTCTTGG			
LoxP3 seq F	TCAATGTATCTTAAGGCGTGACT	60C	600bp	To detect the iMb-Mosaic allele
mTFP1 R	TACTTGGTGAAGGCCCTGTT			
Esm1 Trans F	CTCCGTGCTAAGGGACTCTG	60C	350bp Mutant Band 300 bp Wt band	To detect Esm1 Knock-In
H2B PCR REV	CCTTAGTCACCGCCTTCTTG			
Esm1 WT F	AACAAGAGAGGCTGGCAAGA			
Esm1 WT R	TCCATGCCTGAGACTGTACG			
RR711	GCACTTGCTCTCCCAAAGTC	60C	350bp Mutant Band 250 bp Wt band	To detect Rosa26 Knock-in
RR713	CTTTAAGCCTGCCCAGAAGA			
CAG PCR F	CGGGGTCATTAGTTCATAGCC			
CAG PCR R	CACCTCGACCATGGTAATAGC			
Cdh5 Trans F	GGAGGCTGGAAAGTAGAGCA	60C	550bp	To detect the VE-cadherin(Pac)-CreERT2 allele
CreM R	TCCCTGAACATGTCCATCAG			
RbpjLox F	ATAATTTGCCAAGCCAAAGC	60C	350bp Floxed band 200 bp Wt band	To detect the Rbpj floxed allele
RbpjLox R	GCTCCCCACTGTTGTGAACT			
Dll4 lox (Duarte)	GTGCTGGGACTGTAGCCACT	60C	500bp Floxed band 400 bp Wt band	To detect the Dll4floxed allele
Dll4 lox (Duarte) R	TGTTAGGGATGTCGCTCTCC			
p21f_2B9	ACCCAGCAAAGCCTTGATTCT	60C	600bp KO band 760bp Wt band	To detect the p21 KO allele
NeoF_8B6	CCTTCTATCGCCTTCTTGACGA			
p21r_3B2	CAGGTCGGACATCACCAGGAT			
